# MRA Study on Variation of the Circle of Willis in Healthy Chinese Male Adults

**DOI:** 10.1155/2015/976340

**Published:** 2015-01-05

**Authors:** Chuanya Qiu, Yong Zhang, Caixia Xue, Shanshan Jiang, Wei Zhang

**Affiliations:** Department of Radiology, Civil Aviation General Hospital, Civil Aviation College, Peking University, Beijing 100123, China

## Abstract

*Aim*. To investigate the morphology and variation of the circle of Willis (COW) in healthy Chinese male adults. *Materials and Methods*. We analyzed cerebral magnetic resonance angiography (MRA) images of 2,246 healthy subjects using typical magnetic resonance imaging (MRI) and MRA. 3D-time of flight (TOF) MRA method was applied to all subjects and the classification was therefore achieved according to the integrity level of COW and the developmental situation of vessels. *Results*. The overall incidence of COW integrity was 12.24%, with 7.57% nonvariation integral COW. The incidences of partial integrity and nonintegrity were 70.17% and 17.59%, respectively. The integrity rate of anterior circulation was 78.58%, with a close correlation with A1 segment of the anterior cerebral artery (ACA-A1) developmental condition. The developmental variation rate of ACA-A1 was 28.23% and the variation of the right side was higher than that of the left side. The nonintegrity rate of posterior circulation was 83.93% as the hypoplasia of P1 segment of the posterior cerebral artery (PCA-P1) with an incidence rate of 15.85% for PCA-P1 variation. *Conclusions*. The COW variation is a common phenomenon among the healthy subjects. MRA could enable reflecting the physiological morphology of COW in a comprehensive manner.

## 1. Introduction

The circle of Willis (COW) is an important intracranial collateral circulation system. In patients with cerebrovascular diseases, COW can maintain adequate blood flow and decrease damage of lesion areas through its potential blood redistribution function [1–3]. This compensation depends on the anatomical morphology of COW [4, 5]. The variation of COW can alert cerebral hemodynamics, resulting in various cerebrovascular diseases. In particular, the formation of cerebral aneurysm has correlation with the morphology of COW [6, 7]. Moreover, due to the large variations of COW on the normal population, diverse consequences of clinical disease prognoses are obtained. Previous study of COW was mainly based on autopsy that did not reflect the normal physiological status. With the development of medical imaging, methods like magnetic resonance angiography (MRA) are widely used in clinical research, which enable great progress in the study of the morphology and variation of COW [8, 9]. However, the sample size in previous COW studies on healthy subjects is limited (about 200 subjects). As a noninvasive method without using ionizing radiation, MRA provides a possible survey on massive healthy population. By conducting a retrospective analysis on MRA images of the morphology and variation of COW from large population of healthy subjects (e.g., 2246 subjects in the present study) with normal cerebral MRI, it may confirm the distribution of COW variation types, which will provide anatomical basis for future prognosis and treatment of cerebrovascular diseases.

## 2. Materials and Methods

### 2.1. Participants

2246 Chinese healthy male adults (31–60 y; mean 49.55 ± 3.91 y) were recruited. They had normal or corrected to normal visual acuity and had no history of psychiatric or neurological disorders, with normal brain parenchyma and without the artery stenosis. Based on the MRA images obtained from all participants, the retrospective analysis was carried out on both the morphology of COW and the developmental situation of vessels.

### 2.2. Inspection Methods

All participants underwent a routine MRI and 3D-TOF MRA test using a 1.5T HDX MR machine (GE Company, USA) with 8NVHEAD coil. The scanning series applied on MRI were FSE/T1WI, FSE/T2WI, FLAIR FSE/T2WI, DWI, and GR/T2^*^WI. MRA was adopted using the 3D-TOF MRA, Asset, extended dynamic range, flow compensation, and magnetization transfer techniques with the following parameters: TR 24 m/s, TE 3.1 ms, FA 20°, band width 20.83, FOV 17.6 cm × 20 cm, matrix 192 × 320, and slice thickness 0.6 mm. 156 images were obtained and all images were rebuilt using maximum intensity projections (MIP) and volume rendering (VR) on the ADW4.4 magnetic resonance workstation. These measurements were conducted by a multidimensional analysis, with the diameter of the terminal position of 6 cm for the selected vessel segment (FOV).

### 2.3. Image Analysis

The morphology of COW shown by all images (including original, MIP, and VR images) was analyzed and evaluated according to the existence and developmental state of blood vessels by two experienced neurological radiologists, who engaged in diagnostic neurological radiology for ten years. Consensus was reached after discussion whenever there was a disagreement.

#### 2.3.1. Developmental Situation Determination of ACA-A1 Vessel

**Figure 1:**
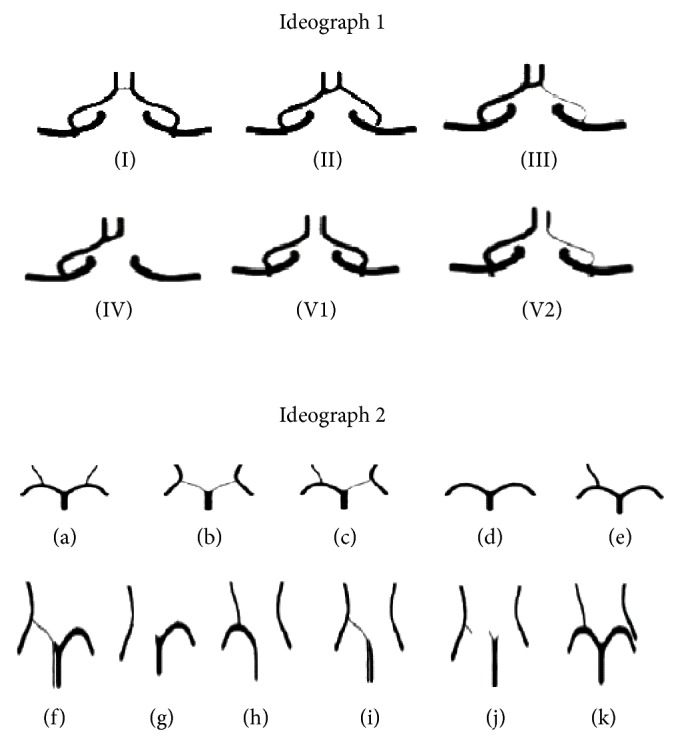
Ideographs 1 and 2 in the present study. Ideograph 1: type I: normal, balanced development of bilateral A1 segment, where ACoA exists; type II: noneven integral type, that is, ACoA exists and unilateral A1 segment is I°; type III: maldevelopment type; type IV: A1 segment is absent. Type V1: A1 segment is evenly developed while ACoA is absent. Type V2: A1 segment is not balanced and ACoA is absent. Ideograph 2: (a) normal posterior circulation; (b) bilateral quasi-FTP type; (c) unilateral quasi-FTP type; (d) bilateral PCoA absent with normal PCA at both left and right rides; (e) unilateral PCoA exists, while contralateral PCoA is absent; (f) unilateral quasi-FTP, with one PcoA absent; (g) unilateral pure FTP, with one PCoA absent; (h) unilateral pure FTP; (i) unilateral pure FTP combining contralateral quasi-FTP; (j) bilateral pure FTP; (k) unilateral duel PCA, extended from internal carotid artery and basilar artery, respectively, without any connection between the two.

For healthy population, the bilateral vessel diameter of the ACA-A1 is larger than 1.5 mm, and the right and left difference are lower than 0.5 mm. In cases of mild variation of vessels, the difference between the left and right vessel diameters of ACA-A1 ranges from 0.5 to 1.0 mm. However, in cases of hypoplasia, the vessel diameter is half less than the normal lateral. MRA image presents no image or nonconsecutive image of ACA-A1 on one side whenever there is any absence of vessels. The classification standard of anterior circulation is based on the existence and developmental situation of ACA-A1 and the existence of ACoA (Figure 1, Ideograph 1).

#### 2.3.2. Classification Standard of PCA

Adult type refers to the fact that PCA-P1 is of bilateral symmetry and the vessel diameter is larger than that of PCoA. FTP type includes four subtypes of the situation when the vessel diameter is less than that of PCoA: (a) type I: there is well-developed PCA-P1 yet the vessel diameter is smaller than that of homolateral PCoA; (b) type II: the vessel diameter is half time less than that of contralateral side and is far less than that of PCoA; (c) type III refers to the situation when PCA-P1 is absent and is formed by PCoA extension; (d) type IV refers to posterior cerebral artery type, with dual PCA extended from basilar artery and internal carotid artery. The classification standard of posterior circulation is based on the existence and developmental situation of PCA-P1 and PCoA and the vessel diameter between them (Figure 1, Ideograph 2).

### 2.4. Statistical Analysis

The *χ* ^2^ test, *χ* ^2^ trend test, and rank-sum test were performed in SPSS version 19, with statistical significance evaluated at the 0.05 alpha level.

## 3. Results

### 3.1. Morphological Variation of COW

**Figure 2:**
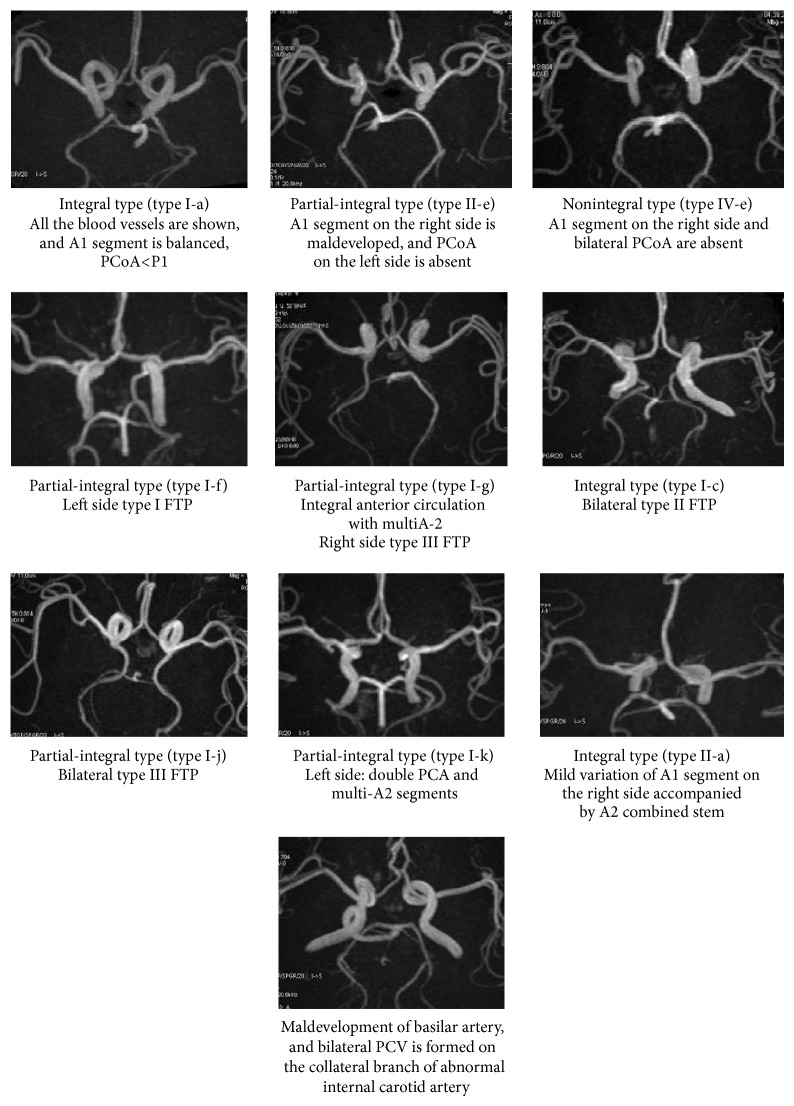
The types of the morphology and variation of Willis' circle (COW). (1) Integral type (type I-a). All the blood vessels are shown, and A1 segment is balanced, PCoA<P1; (2) partial-integral type (type II-e). A1 segment on the right side is hypoplasia, and PCoA on the left side is absent; (3) nonintegral type (type IV-e). A1 segment on the right side and bilateral PCoA are absent; (4) partial-integral type (type I-f). Left side type I FTP; (5) partial-integral type (type I-g). Integral anterior circulation with multi-A2 right side type III FTP; (6) integral type (type I-c). Bilateral type II FTP; (7) partial-integral type (type I-j). Bilateral type III FTP; (8) partial-integral type (type I-k). Left side: double PCA and multi-A2 segments; (9) integral type (type II-a). Mild variation of A1 segment on the right side accompanied by A2 combined stem; (10) hypoplasia of basilar artery. Bilateral PCA is formed on the collateral branch of abnormal internal carotid artery.

Morphological variation of COW was shown in MRA images. Due to the diversity of anterior and posterior circulation morphology, there are more than 10 types of morphological variation. The detailed morphological variation distribution was listed in Table 1 based on the intact degree of anterior and posterior circulation as well as the developmental situation of the ACA-A1 and PCA-P1. It showed that the integral COW occupied 275 cases (about 12.24%). Out of those 275 cases, 105 cases were evident with developmental variation (hypoplasia of posterior circulation or ACA-A1 hypoplasia of anterior circulation); the other 170 (about 7.57%) cases were of normal, developmental, and integral COW structure. 1576 cases (about 70.17%) were observed with partial integral, in which there were 1489 cases with integral anterior circle and nonintegral posterior circle. The other 395 (about 17.59%) cases were of the nonintegral type. The main variations of the present participants were due to type d and type e of posterior circulation (1578 cases; 70.26%). 880 cases (about 55.77%) were of the anterior circulation type (Figure 2).

### 3.2. Morphological Variation of Anterior Circulation

As shown in Table 2, the integrity type rated 78.58% of total participants, in which type I occurred most frequently although types II and III accounted for 29.92% (528/1765) of the integrity anterior circulation. The nonintegrity ratio of anterior circulation was 21.42%, including 29 samples with the obscure ACoA. The balance of the ACA-A1 is of statistical difference with integral anterior circulation, while participants with an unbalanced ACA-A1 possessed a high degree of integral anterior circulation (*χ* ^2^ = 11.578, *P* = 0.001).

As presented in Table 3 about the display of ACA-A1, the proportions of normal development, mild variation, hypoplasia, and the absence of ACA-A1 were 90.87% (2041/2246), 6.01% (135/2246), 2.49% (56/2246), and 0.62% (14/2246) on the left side and 80.90% (1817/2246), 8.82% (198/2246), 6.68% (150/2246), and 3.61% (81/2246) on the right side, respectively. The ACA-A1 in most participants was well balanced and developed and only 634 cases (28.18% of total cases) had ACA-A1 variation. The variations of ACA-A1 were differently distributed on the left and right side (*Z* = 9.944, *P* = 0.000), with apparently higher degree on the right side.

Moreover, variations of ACA-A2 were also found in some subjects, mainly manifesting as 27 cases of ACA-A1 fusion to form single ACA-A2 combined with stem variation. The other 156 cases showed that the abnormal diameter development of the callosum artery formed the third A2 segment. For those with ACA-A1 developmental variation, ACoA in 623 cases was well developed or evident diameter that caused the contralateral ACA-A1 to process blood shunt to ACA-A2 through ACoA. The vessel diameter of ACA-A2 was more obvious than that of ACA-A1. ACoA absence was only found in 11 cases; and the callosomarginal artery was formed on ACA-A2, supplying blood to part of the homolateral callosum area. Contralateral ACA-A1 sent out double ACA-A2 branches, one of which supplied blood to the other areas such as frontal gyrus and cingulate.

**Figure 3:**
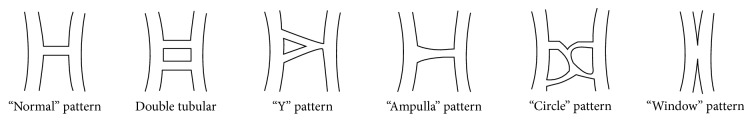
The different shapes of the ACoA.

The morphology of ACoA was also diverse and can be divided into single-branch, double-branch, multibranch, and absence. The shapes were mainly presented as “tubular pattern” or “ampulla pattern,” “Y pattern,” and “window pattern” (Figure 3). As shown in Table 4, the detection rate of ACoA was 81.66% (1834/2246), in which 383 cases were of absence type and 1631 cases were of single-root tubular shape. Other types of variations were rarely observed. ACoA presentation of 29 cases was obscure because of being in close proximity to the bilateral ACA-A2 as well as the resolution ratio of MRA. The patency of ACoA had some relationship with ACA-A1 development and the patency rate of ACoA for ACA-A1 developmental variation in 634 participants was 98.26% (623/634), higher than 76.74% (1237/1612) of those with balanced development of the ACA-A1 (*χ* ^2^ = 148.174, *P* = 0.000).

### 3.3. Morphological Variation of Cerebral Posterior Circulation

Posterior circulation complex variations were classified according to the existence and developmental situation of PCA-P1 and PCoA as well as the diameter between them (Figure 1, Ideograph 2). The most posterior circulations were nonintegral (1885 cases rating 83.93%) presented by type d and type e, while integral posterior circulation only accounted for 16.07% (361/2246; Table 5).

Posterior circulation variation was mainly represented by PCoA variation, manifesting as bilateral/unilateral PCoA, absence, or FTP formation in which PCA-P1 was hypoplasia or absent. The unilateral detection rate of PCoA was 29.92% (672/2246), in which 13.85% (311/2246) was on the left side, 16.07% (361/2246) was on the right, and 21.86% (491/2246) was bilateral. 1083 cases (rating 48.22%) were observed with bilateral PCoA absence. Among all the participants (2246), 438 cases formed FTPs of which 92 cases formed bilateral FTPs and 28 cases were of type IV. As shown in Table 6 about the FTPs distribution of different types, the presentation probability of FTPs on the left was 10.28% and 13.27% on the right side (*χ* ^2^ = 16.200, *P* = 0.003).

Basically, PCA of healthy participants was of adult type. PCA-P1 was maldeveloped or absent at 394 laterals in 356 cases while FTPs were formed, among which 157 cases were of unilateral PCA-P1 hypoplasia, 161 cases of absent unilateral PCA-P1, 10 cases of bilateral hypoplasia, and 28 cases of absent bilateral PCA-P1. The presentations of normal development, hypoplasia, and absence of PCA-P1 were 92.28% (2073/2246), 3.16% (71 out of 2246), and 4.54% (102/2246) for the left, whereas they were 90.16% (2025/2246), 4.72% (106/2246), and 5.12% (115/2246) for the right, respectively. The left and right distributions of PCA-P1 variation were not identical, with higher variation degree on the right (*Z* = 2.576, *P* = 0.01).

### 3.4. The Relationship among FTP Formation, Anterior Circulation Types, and Developmental Situation of the ACA-A1

Among all the participants, there were 295 cases with balanced anterior circulation forming FTPs and 143 cases with unbalanced ACA-A1 forming FTPs. 333 cases showed a mild variation with ACA-A1 forming 70 FTPs, 206 cases had hypoplasia ACA-A1 with 41 FTPs, and 95 cases had absent ACA-A1 with 32 FTPs. The difference between ACA-A1 developmental types of anterior circulation and distribution of PCA types was statistically significant (*χ* ^2^ = 14.165, *P* = 0.003). Meanwhile, as the unbalanced degree of ACA-A1 increased the FTP variation rose (linear-by-linear *χ* ^2^ = 9.188, *P* = 0.002).

The relationship between ACA-A1 variation lateral and FTP was shown in Table 6. FTP was formed on 9.15% (58/634) and 17.03% (108/634) for normal and variation lateral ACA-A1, respectively (*χ* ^2^ = 51.117, *P* = 0.000). There were 15.62% (52/333) with mild variation lateral ACA-A1, 16.50% (34/206) with hypoplasia lateral ACA-A1, and 23.16% (22/95) with absent lateral ACA-A1. As the variation degree of ACA-A1 increased the ratio of the FTP variation side rose (linear-by-linear *χ* ^2^ = 13.340, *P* = 0.000).

### 3.5. Variation between Internal Carotid Artery and Basilar Artery

The persistent trigeminal artery between internal carotid artery and basilar artery was rare. In our study, only 3 cases with persistent trigeminal artery were observed (Figure 2).

## 4. Discussion

### 4.1. Significance of Evaluation of COW Variation by MRA

COW is the most important collateral circulation system and its morphology and function have been studied widely. However, previous studies were mainly based on the study of autopsies, with limitations in reflecting the relationship between COW's morphology and physiological changes of hemodynamic system. Moreover, the number of samples was limited in previous studies and hence their results were not able to represent the population of COW. With the development of modern technology, some new techniques, such as transcranial Doppler (TCD), DSA, and CTA, allow researchers to access the cerebral module on physiological or pathological status. Different from TCD and CTA, MRA (magnetic resonance angiography) is a noninvasive and no-radioactive damage inspection technique, which can be used to undertake a massive study on healthy people on evaluation of COW variation [10–12]. MRA displays an integral COW through different reconstruction methods via one-time collection for further evaluation of the COW in functional manner.

The imaging principle of MRA is to detect blood flow influenced by the diameters of blood vessels. The MT and ZIP technology of 3D-TOF MRA can decrease the saturation effect of blood flow to a large scale, improving the contrast between flowing blood and stationary tissues, so that small blood vessels can be obtained clearly. There was evidence that 3D-TOF MRA can display small blood vessels with the diameter over 0.7 mm [13]. In the present study the MRA resolution ratio was about 0.6 mm and the artery vessels with diameters over 0.6 mm could be displayed by MIP and VR reconstruction. Previous studies showed that when the diameters of ACoA or PCoA were less than 0.7 mm or the ACA-A1 or the PCA-P1 was less than 1.0 mm, the collateral circulation function would not be fully achieved if occluded disease occurred and the effective hemoperfusion in blood supply areas of blocked blood vessels could not be provided [14]. Concerning the pathological function of the COW for providing effective collateral perfusion, when the diameters of the vessels constituting the COW were less than 0.5 mm, the vessels can be regarded as absence. Therefore, the MRA morphology of COW can reflect effectively the physiological cerebral perfusion.

### 4.2. Morphology Classification of COW

Classification of COW is not easy due to its abundant variations. Due to the complex anterior and posterior circulation variation, COW morphology can be divided into dozens of types, aside from the combined types of anterior and posterior circulation. Some researchers [9] divided COW into archetype, modern type, transition type, and combined type from the evolutionary view. However, this classification method ignores the integral morphology of COW, which cannot meet clinical requirements. Krabbe-Hartkamp et al. [14, 15] classified the COW into integrity, partial integrity, and nonintegrity categories based on the MRA image of COW. Integrity refers to the situation when all vessels of the COW are continuously displayed and the diameters are more than 0.8 mm. Partial integrity refers to the fact that only anterior or posterior circulation is integral. However, Krabbe-Hartkamp et al.'s criteria cannot reflect the variation of the COW and the corresponding hemodynamic physiological or pathological changes.

Combining the aforementioned methods, the present study proposed four criteria: (1) the developmental situation of vessels, (2) the difference between left and right vessel diameters, (3) the existence of ACoA and PCoA, and (4) the relationship between the PCoA diameters and PCA-P1. To determine the vessel development situation, according to previous studies [8, 9, 16], we divided ACA-A1 into four types. For a mild variation type of ACA-A1, the difference between the diameters of left and right ACA-A1 was between 0.5 mm and 1.0 mm. This study observed that if the diameter difference between the left and the right sides was over 0.5 mm, the blood flow redistribution between the left and right sides of anterior circulation might occur, manifesting as the beginning diameter of ACA-A2 on the variation lateral which was larger than that of the variation lateral ACA-A1. Previous study showed that the change of hemodynamics plays an important role in the formation of cerebral aneurysms, homolateral cerebral infarction, and arteriosclerosis [16–21]. The ratios of mild variation, hypoplasia, and absence of ACA-A1 were 8.82%, 6.68%, and 3.61% for right side and 6.01%, 2.49%, and 0.60% for left side, respectively. Among all participants, there were 634 cases showing a congenital development variation of the ACA-A1. The congenital variation of ACA-A1 displayed left-right side difference, where the variation of ACA-A1 on the right side was more evident (*P* < 0.01), indicating a dominant position of left development. This kind of situation may be related to right handedness with a dominant and superior position on left cerebral hemisphere [22]. Though the classification of the COW was complex, this classification reflects the true relationship between the morphology and hemodynamics of cerebral vessel.

Among the COW of 2246 participants, the integrity ratio of anterior circulation was 78.58% and the variation ratio of types II and III reached to 23.51%. The cases rating of 83.93% (1885) with posterior circulations mainly manifested as nonintegral and the integrity rate was only 16.07% including 131 cases with FTPs. Another 28 cases had type K posterior cerebral artery which was rarely reported in the literature and, maybe, this variation only occurs in Chinese people.

The proportions of integrity, partial integrity, and nonintegrity of the COW were 12.24%, 70.17%, and 17.59%, respectively. There were 170 cases of the integral type showing a symmetrical blood development, while there were 1490 cases predominating in the partial-integral type with an integral anterior circle but nonintegral posterior circle. The low ratio of the integral COW was caused by the low ratio of integral posterior circulation, as the unilateral/bilateral absence of PCoA (PCoA absence of type d and type e: 70.26%). The ratio of the integral COW was lower than that reported in previous literature [8, 9, 23]. Riggs and colleagues reported that the ratio of integral COW of anatomy samples observed by naked eye was 21%; however, Alpers and colleagues reported the ratio of integral COW of 52%, including those dissymmetry lumens possessing a diameter over 1 mm. Compared with Krabbe-Hartkamp et al.'s study relevant to the COW with MRA [15], the present study showed the lower ratio between the integral posterior circulation and COW. The reason could be spatial resolution limitation and indeed small blood vessels in autopsies cannot be displayed by MRA. In physiological status, if the blood pressure difference between anterior and posterior circulation is small, distributary from the front to rear is not evident, which cannot be presented by MRA. This discrepancy between Krabbe-Hartkamp's study and the present findings could be related to the selected sequence and parameters. Moreover, the number of samples was small (150 cases) in Krabbe-Hartkamp's study.

### 4.3. The Relationship between the Variation of ACA-A1 and ACoA Patency and the Formation of FTP

ACoA was usually patent when ACA-A1 mutated, with the patency rate of 98.26% for 634 subjects with ACA-A1 variation, which was higher than 76.74% of those with balanced development of ACA-A1 (*P* < 0.01). The variation of ACA-A1 resulted in a decrease of blood supply to the far-end of ACA-A2 dominated area which failed to meet the requirement of cerebral tissue perfusion. We presume that, in order to fulfill the requirement of cerebral tissues perfusion, the diameter of the contralateral ACA-A1 and ACoA patency is promoted in the embryonic development period. The measurements of blood vessel diameters in this study demonstrated that the larger difference of ACA-A1 development between two sides would result in larger diameter of ACoA. Among 634 cases with variation of ACA-A1, only 11 cases showed ACoA absence, combined with maldeveloped ACA-A2 at the same time. In such situation, double-branch ACA-A2 blood vessels were extended from the contralateral ACA-A1 to ACA-A2 dominated area on both sides after running along the longitudinal segmentation for a short distance.

We also found that more FTPs were observed on the right than on the left side and interestingly, among the formed FTPs, 217 subjects formed lateral pure FTPs while 28 subjects formed bilateral pure FTPs. Some researchers [24, 25] considered that pure FTPs might have prevented the communication between anterior and posterior circulation and collateral circulation intermeningeal artery and consequently collateral circulations of carotid artery and basilar artery would decrease. For patients with FTP, the blood supply function of collateral artery was insufficient when artery thrombosis occurred, with the infarction area of carotid artery thrombosis appearing to be much larger. For patients with cerebral hernia, the risk of massive cerebral infarction was higher. Our study found that FTPs were closely correlated to the developmental situation of ACA-A1. According to the process of cerebral artery embryo development, we considered that the development of carotid artery systems of cerebral artery is earlier than that of the basilar artery system and the developed PCoA inevitably influences the development of the PCA-P1 of the corresponding lateral of basilar artery. If one lateral side of ACoA is maldeveloped or is absent, the blood supply to PCoA from the homolateral internal carotid artery may impel the PCoA of this developed lateral. Therefore, the diameter of PCoA can grow larger than PCA-P1 from basilar artery or even cause PCA-P1 absence and then form quasi-FTP or pure FTP.

Before concluding, we should reiterate two procedural decisions that constrain the interpretation of the present findings. First, only male participants were recruited and the gender effect needs to be investigated in the future. Second, the display of small blood vessels should be improved based on the higher spatial resolution of MRI.

These limitations notwithstanding, different from previous studies using small samples [26, 27], the present study recruited 2246 participants and increased the reliability and representativeness of the result so to present the basic situation and variation of the COW in the healthy male Chinese. The establishment of the preliminary cerebral artery formation module in healthy person can play an important role in the clinical prognosis of cerebrovascular diseases.

## Figures and Tables

**Table 1:** Morphological variation of COW.

Type	Integral posterior circulation			Nonintegral posterior circulation							
	a	b	c	d	e	f	g	h	i	j	k
Integral anterior circle											
I	143	16	44	593	287	47	31	34	17	8	17
II	27	3	17	160	70	12	10	11	6	2	7
III	16	0	9	109	40	10	8	5	1	2	3
Nonintegral anterior circle											
IV	2	2	7	43	18	10	5	2	2	3	1
V1	42	6	27	159	93	9	7	9	11	12	
V2					2	4	2	2			1

**Table 2:** Classification of anterior circulation.

Type	Integral	Nonintegral
A1 balanced type	1237 (I)	375 (V1)
A1 unbalanced type	528 (II, III)	106 (IV, V2)

Note: *χ* ^2^ = 11.578, *P* = 0.001.

**Table 3:** Developmental variation of the A1 segment.

A1 segment	Normal (side)	Variation (side)			Mean rank sum
		Mild variation	Hypoplasia	Absence	
L	2041	135	56	14	2130.10
R	1817	198	150	81	2362.90

Note: *Z* = 9.944, *P* = 0.000.

**Table 4:** Morphology and number variation of ACoA.

ACoA	Normal	Double-branch	Y pattern	Window pattern	Ampulla	Circle pattern	Absence	Not clear
Cases	1631	76	22	88	14	3	383^*^	29

^*^Including 27 cases of A2 segment combined stem.

**Table 5:** Posterior circulation developmental morphology.

Types	Integral posterior circulation			Nonintegral posterior circulation								Total proportion
	a	b	c	d	e	f	g	h	i	j	k	
Cases	230	27	104	1066	512	90	63	61	37	28	28	16.07%

**Table 6:** The relationship between normal lateral and variation lateral and the formation of homolateral embryonal pattern of 634 cases with A1 segment variation.

Developmental situation of A1 segment	PCA type (side)					FTP ratio (%)
	Normal	I	II	III	IV	
Normal	576	8	16	13	21	9.15
Mild variation	281	5	20	23	4	15.62
Hypoplasia	172	1	15	16	2	16.50
Absence	73	2	7	12	1	23.16

Note: *χ* ^2^ = 51.117, *P* = 0.000; linear-by-linear *χ* ^2^ = 13.340, *P* = 0.000.
